# Decreased DMI sensitivity of *Plenodomus biglobosus* (phoma of oilseed rape) associated with CYP51 substitution G476S

**DOI:** 10.1002/ps.8926

**Published:** 2025-05-28

**Authors:** Kevin M King, Liza M González‐Rodríguez, Joanna Kaczmarek, Małgorzata Jędryczka, Jonathan S West

**Affiliations:** ^1^ Rothamsted Research Harpenden United Kingdom; ^2^ Institute of Plant Genetics Polish Academy of Sciences Poznan Poland

**Keywords:** *Erg11*, fungicides, *Leptosphaeria biglobosa*, *Leptosphaeria maculans*, mode of action, oilseed rape

## Abstract

**BACKGROUND:**

Phoma leaf spot/stem canker is an international oilseed rape (*Brassica napus*) disease caused by *Plenodomus lingam* (*Pl*) and *Plenodomus biglobosus* (*Pb*). Phoma management can include fungicide applications, often sterol 14α‐demethylase (CYP51) inhibitors (DMIs). *Pl* and *Pb* isolates (collected throughout Poland in 2024) were screened for *in vitro* sensitivity to the DMI prothioconazole‐desthio, and molecular mechanisms associated with altered sensitivity were investigated.

**RESULTS:**

*Pl* isolates (2024) were less sensitive (4‐fold) than older (before 2002) isolates. All *Pl* (2024) isolates carried *CYP51* promoter inserts, likely a *Sahana* transposable element (TE) fragment, previously associated with *CYP51* overexpression and decreased DMI sensitivity. In addition, *Pb* isolates with decreased DMI sensitivity were also identified, but without such TE inserts. *CYP51* gene sequencing instead revealed substitution G476S that was absent in more‐sensitive G476 isolates. G476S homologues have been linked to decreased DMI sensitivity in multiple other fungi. *Pb* G476S isolates were less sensitive (7.3‐fold) than *Pb* G476, widespread throughout Poland, and comprised 14 of 24 (58%) *Pb* isolates tested. Although *CYP51* promoter inserts were detected in 2 of 24 (8%) *Pb* isolates, these were promoter sequence duplications (not TEs) and not obviously associated with decreased sensitivity. *Pl* isolates carrying promoter inserts (predominant in recently tested European populations) were more sensitive (2.6‐fold) than *Pb* G476S, but less sensitive (2.8‐fold) than *Pb* G476.

**CONCLUSION:**

We provide first evidence for a CYP51 substitution associated with decreased DMI sensitivity in *Pb*. Result implications for phoma management, how DMI usage might influence pathogen population structure, resistance management strategies and future research required are discussed. © 2025 The Author(s). *Pest Management Science* published by John Wiley & Sons Ltd on behalf of Society of Chemical Industry.

## Graphical abstract

Here, we report for the first time a target site mutation in the *CYP51* gene of the oilseed rape phoma stem canker fungus *Plenodomus biglobosus* (*Leptosphaeria biglobosa*) that is associated with decreased sensitivity to sterol demethylation inhibitor fungicides.

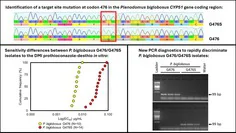

## 1 INTRODUCTION

Phoma leaf spot and stem canker (blackleg) is an internationally important threat to oilseed rape (OSR; *Brassica napus*) production. Two main ascomycete fungi are responsible for the disease – *Plenodomus lingam* (synonym *Leptosphaeria maculans*) and *Plenodomus biglobosus* (syn. *Leptosphaeria biglobosa*).^1^, ^2^ A third species has recently been described, *Plenodomus dezfulensis*, that is most closely related to *P. biglobosus* but is not so far considered to be of economic importance having been reported on only a small number of leaves in Iran.^3^ The disease is primarily caused by the ascospores of the pathogen, which can be detected using microscopic and molecular tools.^4^, ^5^ Moreover, the air‐transported ascospores may contribute to autumn asthma in areas of oilseed rape production.^6^ Back‐trajectory modelling and DNA‐based species‐specific detection methods allow tracking of the spore transport of *Plenodomus* spp. in air masses.^7^ *P. lingam* and *P. biglobosus* co‐occur in many parts of the world, including the internationally important OSR‐growing regions of Australia, Canada and Europe,^8^ although in some parts of the world only one or other of the species are known to occur (e.g. *P. biglobosus* in China^9^) or at least appear predominant (e.g. *P. lingam* in South Africa^10^). Development of the pathogens and the subsequent yield loss strongly depend on weather and are greater after warm but wet autumns.^11^, ^12^

*P. lingam* has traditionally been considered the economically important OSR pathogen, being more often associated with damaging basal stem cankers, compared with *P. biglobosus* that has been linked to less‐damaging upper stem lesions.^8^ Research into phoma disease management, however, has focused predominantly on *P. lingam*, with the deployment of host resistance genes targeted to this species that are not effective against *P. biglobosus*; Huang *et al*.^13^ suggest that effective control of *P. lingam* has led to selection for *P. biglobosus*, which is increasingly problematic in the United Kingdom (UK).

Application of fungicides, predominantly sterol demethylation inhibitors (DMIs; FRAC code 3) is the other key phoma disease management strategy. In the UK in 2023 alone, >500 000 ha of crop were treated with this mode of action (MoA) chemistry (nearly two applications per crop).^14^ Similarly, studies performed in 2023 in Poland by KYNETEC, an agricultural market research company, have demonstrated that the area of oilseed rape treated with DMIs exceeded 1.93 million hectares, which means that, on average, each field was treated twice or more times per season (www.kynetec.com/pl). A recent 2025 study indicates that, in Poland, environmental contamination with the DMI tebuconazole is due to use of this compound in agriculture for disease control, which runs off into surface water following application.^15^

Decreased DMI sensitivity is often mediated either through alterations in the structure of the target *CYP51* (encoding the sterol 14 alpha‐demethylase) or overexpression of *CYP51* that is typically mediated by insertions into the upstream promoter region; increased fungicide efflux via overexpression of efflux pumps is another possibility although other resistance mechanisms are also known to occur.^16^ In 2015, *P. lingam* isolates with decreased DMI sensitivity were identified for the first time in Australia,^17^ and subsequently in eastern and western Europe,^18^, ^19^, ^20^ and that has been associated with *CYP51* promoter inserts and target site overexpression.^21^ To date, *CYP51* promoter inserts have not been identified for *P. biglobosus*, nor have *CYP51* target site mutations associated with decreased DMI sensitivity been reported for either species,^19^, ^22^, ^23^, ^24^ although ongoing monitoring is required for early detection of these resistance mechanisms should they emerge in future.

In the majority of European *P. lingam* populations tested to date (including the Czech Republic, Germany, Ireland and the UK; although not yet detected in France), such *CYP51* promoter inserts, typically transposable elements (TEs), have now been reported at high frequencies of >85%, with a 237 bp fragment of the *Sahana* TE the predominant variant.^19^, ^20^ These findings, combined with the fact that King *et al*.^19^ report some evidence that *P. lingam* isolates (carrying *CYP51* promoter inserts) were less sensitive to DMIs *in vitro* than *P. biglobosus* (no inserts) to DMIs, suggest that application of this MoA might lead to future selection for *P. lingam*. Eckert *et al*.^25^ suggest that differential sensitivity to fungicides might affect the *P. lingam*/*P. biglobosus* population structure. Effective control of phoma leaf spotting and stem canker, including the use of fungicides, thus requires targeting of both *P. lingam* and *P. biglobosus*,^13^ and should be underpinned by good understanding of the population biology of both pathogens.

The main aims of this study were first, to screen populations of *P. lingam* and *P. biglobosus* sampled in 2024 from Poland (a country in central‐east Europe for which such recent data are currently unavailable) to investigate the possibility of decreased DMI sensitivity for either species *in vitro*; second to investigate the underpinning molecular mechanisms associated with any sensitivity shifts identified; and third to assess whether there are differences in DMI sensitivity between the species that might impact on the pathogen population structure. This paper reports the findings of these studies, and considers their implications in terms of phoma stem canker management.

## 2 MATERIALS AND METHODS

### 2.1 Fungal isolation, DNA extraction and species‐specific polymerase chain reactions

Stems of winter OSR exhibiting clear phoma stem canker lesions were collected from multiple sites throughout Poland in July and August 2024. Fungal isolation was attempted from stems within 1 week of collection (for a very small number within 2 weeks), predominantly from basal cankers. From each stem, the outer lesioned material was removed using a sterile scalpel, and a small fragment of the underlying necrotic material (~3mm^2^) was excised. These fragments were surface sterilised for 1 min in 5% sodium hypochlorite solution (v/v), rinsed twice in sterile distilled water, and lastly placed on sterile filter paper in a laminar flow hood until dry. Lesion segments were transferred to potato dextrose agar (PDA) plates (containing penicillin and streptomycin at final concentrations of 50 units mL^−1^ and 50 μg mL^−1^, respectively; Life Technologies Corporation, Grand Island, New York, USA) and incubated at 18 °C for ~1 week until colonies were evident. Subsequently, a small piece of mycelium was taken from each colony margin using a sterile needle and used to establish a pure isolate. Isolates were each obtained from different stems; i.e., one isolate was retained per individual stem. All isolates obtained from single mycelial tips were maintained in duplicate at 4 °C on PDA slopes at Rothamsted Research (Hertfordshire, UK). In addition to these newly collected isolates, five older Polish isolates (LmPL002, 012, 014, 016, 020) collected before 2002 were also sourced from the OREGIN culture collection that had been maintained at 4 °C on PDA slopes at Rothamsted Research.

DNA was extracted and species identities of obtained isolates confirmed as either *P. lingam* or *P. biglobosus* via multiplex species‐specific polymerase chain reaction (PCR) analysis,^26^ as described previously.^19^, ^27^ In total, 37 isolates of *P. lingam* collected from 12 sites and 24 isolates of *P. biglobosus* from 11 sites (Table 1) were newly collected from Poland in 2024 for this study. The distribution of the isolates of both species from Poland is shown graphically in Fig. 1.

**Table 1:** Details of 24 *Plenodomus biglobosus* isolates collected from oilseed rape crops in Poland in 2024 with information on *in vitro* EC_50_ sensitivity values to the sterol demethylation inhibitor prothioconazole‐desthio and *CYP51* genotype

Isolate code^†^	Geographic origin		Mean EC_50_ (μg mL^−1^): prothioconazole‐desthio^‡^	*CYP51* promoter insert detected	*CYP51* gene genotype based on sequencing (GenBank accession number)^§^	*CYP51* gene genotype (PCR diagnostics)^¶^
	Place	Voivodship				
24–39‐9	Suchodębie	Lodzkie	0.004	No	G476 (PV013619)	G476
24–39‐5	Suchodębie	Lodzkie	0.005	No	G476 (PV013620)	G476
24–48‐9	Mózgowo	Warmia‐Masuria	0.005	No	G476 (PV013621)	G476
24–51‐1	Krościna Mała	Lower Silesia	0.005	No	G476 (PV013622)	G476
24–41‐2	Gole	Kuyavia‐Pomerania	0.006	No	—	G476
24–43‐5	Mierzewo	Greater Poland	0.006	No	—	G476
24–50‐4	Czemice Borowe	Mazovia	0.006	No	G476 (PV013623)	G476
24–41‐1	Gole	Kuyavia‐Pomerania	0.007	No	G476 (PV013624)	G476
24–35‐2	Wola Wielka	Subcarpathia	0.008	No	G476 (PV013625)	G476
24–43‐6	Mierzewo	Greater Poland	0.010	No	G476 (PV013626)	G476
24–44‐3	Dębowo	Pomerania	0.014	No	G476S (PV013627)	G476S
24–43‐7	Mierzewo	Greater Poland	0.021	No	G476S (PV013628)	G476S
24–51‐3	Krościna Mała	Lower Silesia	0.025	No	G476S (PV013629)	G476S
24–46‐3	Gruszów	Upper Silesia	0.033	No	—	G476S
24–46‐10	Gruszów	Upper Silesia	0.035	No	—	G476S
24–46‐9	Gruszów	Upper Silesia	0.036	No	—	G476S
24–46‐1	Gruszów	Upper Silesia	0.038	No	—	G476S
24–42‐9	Hrubieszów	Lublin	0.041	No	—	G476S
24–44‐6	Dębowo	Pomerania	0.046	336 bp insert	G476S (PV013630)	G476S
24–43‐1	Mierzewo	Greater Poland	0.047	No	G476S (PV013631)	G476S
24–42‐6	Hrubieszów	Lublin	0.049	No	—	G476S
24–38‐4	Radostowo	Pomerania	0.063	336 bp insert	G476S (PV013632)	G476S
24–42‐4	Hrubieszów	Lublin	0.076	No	—	G476S
24–51‐4	Krościna Mała	Lower Silesia	0.089	No	G476S (PV013633)	G476S

EC_50_, effective concentration 50; PCR, polymerase chain reaction.

^†^ Isolates confirmed as *P. biglobosus* based on species‐specific multiplex PCR.^26^

^‡^ Based on two technical replicates.

^§^ Based on sequencing of the *CYP51* gene coding region.

^¶^ Isolates typed as either G476 or G476S using PCR primer sets PbCommon2/PbGly2 or PbCommon2/PbSer2, respectively.

**Figure 1:**
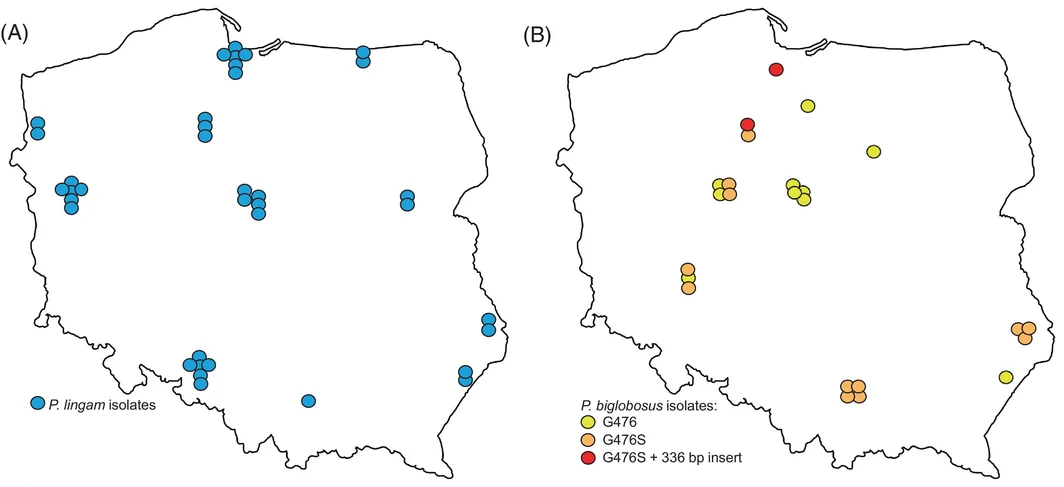
Geographical distribution of (A) 37 *Plenodomus lingam* and (B) 24 *Plenodomus biglobosus* isolates collected from oilseed rape in Poland in 2024 used in this study. All *P. lingam* isolates shown in (A) contained *CYP51* promoter inserts. The *CYP51* genotype of the *P. biglobosus* isolates in (B) is indicated in the legend.

### 2.2 Fungicide sensitivity testing

*In vitro* prothioconazole‐desthio sensitivity was tested for: 37 *P. lingam* isolates collected from Poland in 2024; 24 *P. biglobosus* isolates collected from Poland in 2024 (Table 1); and 5 *P. lingam* isolates collected from Poland before 2002. Testing was as described in King *et al*.,^19^ except that isolates were incubated for an additional 48 h under near‐ultraviolet light to ensure sufficient sporulation before use. To account for minor between‐run variation in sensitivity testing, a simple conversion factor was calculated so that the combined mean effective concentration 50 (EC_50_) value of the five older (before 2002) reference Polish *P. lingam* isolates used in this study (lacking *CYP51* promoter inserts) was 0.004 μg mL^−1^ (the value obtained for isolates lacking inserts by King *et al*.^19^). Subsequently, the entire EC_50_ data set of this study was uniformly scaled using this calculated conversion factor (all data remained proportional). This uniform adjustment of raw EC_50_ values ensured consistency between data sets obtained in this study and King *et al*.^19^

### 2.3 Molecular analyses

#### 2.3.1 PCR screening and sequencing of *P. lingam* isolates *CYP51* promoter inserts

The promoter region of the *CYP51* gene was PCR amplified for 37 *P. lingam* isolates collected from Polish OSR crops in 2024 using primers EPS1/EPS6.^21^ In addition, these primers were also applied to DNA extracted from the five older (collected before 2002) Polish *P. lingam* isolates. PCR was carried out in 25‐μL volumes, containing 12.5 μL of RedTaq ReadyMix (2× concentrate; St Louis, Missouri, USA), 0.2 μL each of primers EPS1 and EPS6 (100 μm stocks) (EPS1: 5′‐AGCACCCATGGACCACGG‐3′; EPS6: 5′‐CAGGATAAAGGAGGCGAAG‐3′), 10.1 μL of PCR‐grade water and 2 μL of genomic DNA (~20 ng total). Reaction conditions were: 35 cycles of 95 °C for 1 min, 60 °C for 1 min, 72 °C for 1 min; a final elongation step of 72 °C for 5 min; and a final hold at 4 °C. PCR amplicons were visualized by electrophoresis on 3% agarose gels. Two reference *P. lingam* isolates, one without a *CYP51* insert and one carrying the 237 bp insert predominant in most contemporary European pathogen populations tested to date, were also screened.^19^ PCR amplicons were visualised by agarose gel electrophoresis and the presence or absence of inserts confirmed by amplicon size comparison with the reference isolates. Amplicons obtained for two representative *P. lingam* isolates (24‐42‐2, 24‐50‐7) were purified using a MinElute PCR purification kit (Qiagen, Strasse 1 40724 Hilden, Germany) and sent to GeneWiz UK Ltd for bidirectional sequencing. Sequences were aligned and analysed using Geneious software v. 8.1 (Biomatters Ltd., Auckland, New Zealand). *CYP51* promoter inserts present were described, as is convention,^28^ based on the position of the insert relative to the gene start codon, followed by the size of the insert. Because inserts were known to include a few base pairs of sequence duplicated from the *CYP51* promoter region, the positioning of the inserts relative to the start codon was based on the downstream positioning of this repeated sequence.^19^

#### 2.3.2 Sequencing of the *CYP51* gene for *P. biglobosus* isolates

The *CYP51* gene was amplified and sequenced for 15 *P. biglobosus* isolates (Table 1). Primers used for amplification of the entire *P. biglobosus CYP51* gene coding region were designed in this study, and were based on available genome sequence of this species (GenBank accession FO905635, positions spanned 23 694 to 25 479).^29^ Primers were designed to amplify the entire *CYP51* gene (1642 bp that includes a 58 bp intron), with outer primers designed to target the gene flanking regions (predicted PCR amplicon size 1786 bp). PCR was carried out in 50‐μL volumes, containing 5 μL of 10× PfuUltra II reaction buffer, 0.5 μL of dNTP mix (25 mm stock), 0.15 μL each of outer primers LbigCYP51geneF_1 (5′‐AAGTCTCTCGTTCTTCCGCG‐3′) and LbigCYP51geneR_1 (5′‐AGCCAGCCATTCCATCAAGG‐3′) (each 100 μm stocks), 40.7 μL of PCR‐grade water, 1 μL of PfuUltra II Fusion HS DNA polymerase (Agilent Technologies Inc.) and 2.5 μL of genomic DNA (25 ng total). Reaction conditions were as follows: 35 cycles of 95 °C for 20 s, 60 °C for 20 s, 72 °C for 1 min; a final elongation step of 72 °C for 3 min; and a final hold at 4 °C. Amplicons were sequenced using primers LbigCYP51geneF_1 and LbigCYP51geneR_1, plus an additional two internal primers LbigCYP51int1 (5′‐TGGATGACGACGAGTGTTGG‐3′) and LbigCYP51int2 (5′‐CGCAGCAAAGAAAGACTCACA‐3′). Sequences were aligned and inspected for the presence of synonymous or non‐synonymous mutations. Molecular analyses revealed that some of the *P. biglobosus* isolates sequenced had no CYP51 substitution at codon 476 (were wild‐type, hereafter referred to as G476 isolates), whereas others carried substitution G476S (hereafter referred to as G476S isolates); the numbering here is based on the nomenclature of Mair *et al*.^30^ and by reference to the *Zymoseptoria tritici* archetype (GenBank accession AY253234).

#### 2.3.3 PCR diagnostics for the detection/discrimination of *P. biglobosus* G476/G476S isolates

Two new diagnostic assays, developed to be used individually in two different sets of reactions, were designed to target the non‐synonymous mutation at *CYP51* codon 476 [G476: GGT; G476S: AGT]. For both primer sets, the forward ‘common’ primer was the same (PbCommon2: 5′‐CCGACGAAAAGGACGACGAG‐3′). For G476 isolates the specific reverse primer was PbGly2 (5′‐AGCGATGTCTTCCTGCACC **G**‐3′); for G476S isolates the specific reverse primer was PbSer2 (5′‐AGCGATGTCTTCCTGCACT **G**‐3′.) In each of the specific reverse primers, the underlined base represents the key SNP targeted, whereas the final bold base is an intentional mismatch introduced to improve assay specificity. Primers were designed to amplify a product of 99 bp. Details of the *P. biglobosus* isolates used for validation of the developed assays are given in Table 1, although *P. lingam* DNA (isolate 24‐40‐4) was also screened as a further test of specificity. Isolates were considered G476 if an amplicon of the expected size was produced using primer pair PbCommon2/PbGly2 but not PbCommon2/PbSer2, and G476S if the opposite result was found. PCR analysis was carried out in 25‐μL volumes, containing 12.5 μL of RedTaq ReadyMix (2× concentrate), 0.2 μL each of the forward and reverse primers (100 μm stocks), 10.1 μL of PCR‐grade water and 2 μL of genomic DNA (~20 ng total). Reaction conditions were: 35 cycles of 95 °C for 1 min, 66 °C for 1 min, 72 °C for 1 min; a final elongation step of 72 °C for 5 min; and a final hold at 4 °C. PCR amplicons were visualized by electrophoresis on 2% agarose gels.

#### 2.3.4 Screening of *P. biglobosus* isolates for *CYP51* promoter inserts

All 24 *P. biglobosus* isolates (Table 1) were screened for *CYP51* promoter inserts using primers KK2F/R, as previously described in King *et al*.^19^ Amplicons obtained for three isolates (24‐38‐4, 24‐44‐3, 24‐44‐6) were purified, bidirectionally sequenced and analysed in Geneious.

### 2.4 Statistical analyses of data sets

All data were collated in MS Excel, where initial exploratory data analysis and graph production were carried out. Subsequent statistical analyses mostly used the GraphPad Prism software package v. 8.4.2 (San Diego, California, USA).

#### 2.4.1 *P. lingam* data set

Analyses of EC_50_ data suggested some violations of the assumption of normality (Kolmogorov–Smirnov tests) and equal variance (Levene test) (data not shown). Thus, non‐parametric Kruskal–Wallis one‐way analysis of variance (ANOVA) tests were used to explore whether significant differences could be identified between six *P. lingam* populations: Poland (isolates collected before 2002, *N* = 5), Poland (2024, *N* = 37), France (2023, *N* = 3), the UK (collected 2022/2023, *N* = 34), Ireland (2023, *N* = 9) and Germany (2022/2023, *N* = 9). Data for the two Polish populations tested were obtained in the current study; data for the other four populations were sourced from King *et al*.^19^ If significant differences were identified, *post hoc* Dunn's multiple comparisons tests, commonly used following Kruskal–Wallis one‐way ANOVA and that make no assumptions as to whether the data are normally distributed, were subsequently carried out to pinpoint between which populations differences occurred.

#### 2.4.2 *P. biglobosus* data set

Analyses of EC_50_ data suggested violation in the assumption of equality of variance (Levene test) but not normality (Kolmogorov–Smirnov test); thus Welch's *T*‐test was used to determine whether significant differences could be identified between G476 (*N* = 10) and G476S (*N* = 14) isolates.

#### 2.4.3 *P. lingam versus P. biglobosus* data sets

Analyses of EC_50_ data suggested violation in the assumption of equality of variance (Levene test) but not normality (Kolmogorov–Smirnov test); thus Welch's one‐way ANOVA test was used to determine whether significant differences could be identified between isolates (all collected in 2024 from Poland) of *P. lingam* carrying *CYP51* promoter inserts (*N* = 37), *P. biglobosus* G476 (*N* = 10) and *P. biglobosus* G476S (*N* = 14). If significant differences were identified, *post hoc* Games–Howell multiple comparisons tests, used because of the violation of the assumption of equality of variance, were subsequently carried out to pinpoint between which groups differences occurred.

## 3 RESULTS

### 3.1 *P. lingam* sensitivity testing and molecular analyses

#### 3.1.1 *In vitro* sensitivity testing

Screening for prothioconazole‐desthio sensitivity showed that the six groups of *P. lingam* isolates, ranked from the lowest to the highest median EC_50_ values with the interquartile range (IQR) given in parentheses, were from: France (collected 2023, *N* = 3), EC_50_ 0.003 (0) μg mL^−1^; Poland (isolates collected before 2002, *N* = 5), EC_50_ 0.004 (0.003) μg mL^−1^; the UK (collected 2022/2023, *N* = 34), EC_50_ 0.014 (0.010) μg mL^−1^; Ireland (collected 2023, *N* = 9), EC_50_ 0.015 (0.015) μg mL^−1^; Poland (collected 2024, *N* = 37), EC_50_ 0.016 (0.006) μg mL^−1^; and Germany (collected 2022/2023, *N* = 9), EC_50_ 0.019 (0.010) μg mL^−1^ (Fig. 2). Kruskal–Wallis test one‐way ANOVA results suggested significant differences between one or more pairs of the six groups (*H* [5] = 26.25, *P* < 0.01). *Post hoc* comparisons using Dunn's multiple comparisons test showed that significant differences (at least *P* < 0.05) occurred between the more‐sensitive isolates from both Poland (collected before 2002) and France *versus* less‐sensitive isolates from Germany, Ireland, Poland (collected 2024) and the UK (collected 2022/2023). No other significant differences were observed in any pairwise combinations, apart from between the UK and slightly less‐sensitive German isolates for which small, but statistically significant differences were detected. These results indicate sensitivity shifts (fourfold) between more‐sensitive older (collected before 2002) and less‐sensitive recent (collected 2024) Polish *P. lingam* isolates.

**Figure 2:**
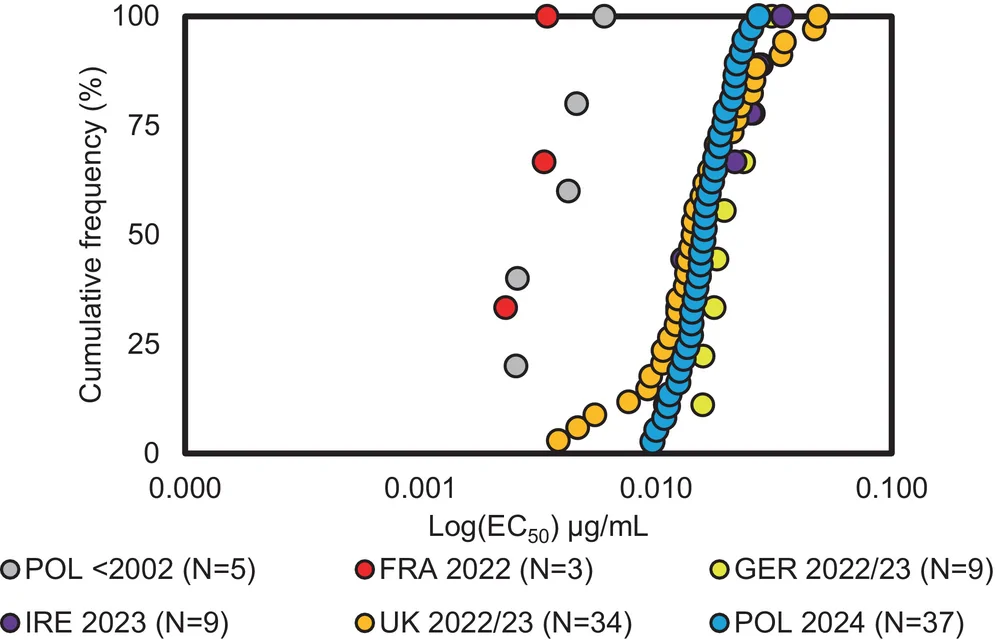
*In vitro* sensitivity of *Plenodomus lingam* (all carrying *CYP51* inserts) isolates from France (FRA), Germany (GER), Ireland (IRE), the United Kingdom (UK) and Poland (POL) to the sterol demethylation inhibitor (DMI) prothioconazole‐desthio. The years that isolates were collected are indicated in the legend (i.e. ‘22/23’ indicates 2022–2023). Isolates are ranked according to increasing effective concentration 50 (EC_50_) values (cumulative). The number of isolates in each group is indicated (*N*). Each data point shown represents the mean of two technical replicates.

#### 3.1.2 Molecular screening for *CYP51* promoter inserts

Screening via PCR of all 37 Polish *P. lingam* isolates collected in 2024 using primers EPS1/EPS6 revealed that all carried *CYP51* promoter inserts (Fig. 1(A)). The five *P. lingam* isolates (collected before 2002) all lacked promoter inserts (data not shown). Based on visual inspection of amplicons resolved by agarose gel electrophoresis, almost all (36 of 37; 97%) appeared to carry inserts that, based on visual inspection of amplicons, were of an identical, or very similar size, to that of a reference *P. lingam* isolate carrying the −99, ins.237 insert. Sequencing of amplicons of one such representative *P. lingam* isolate (24‐50‐7) confirmed 100% identity to GenBank accession OR961466, a *P. lingam* isolate with a 237 bp *Sahana* TE insert at 99 bp upstream of the start codon. A single isolate (1 of 37; 3%) gave a larger amplicon in PCR screening (24‐42‐2) and sequencing of this isolate confirmed 100% identity to GenBank accession OR961467, a *P. lingam* isolate previously confirmed by King *et al*.^19^ to carry a 360 bp insert at 382 bp upstream of the start codon.

### 3.2 *P. biglobosus* sensitivity testing and molecular analyses

#### 3.2.1 *In vitro* sensitivity testing

Initial visual inspection of sensitivity data revealed two distinct groups. The first group comprised ten isolates that appeared relatively sensitive to prothioconazole‐desthio; molecular analyses (discussed in more detail below) revealed no CYP51 substitutions including at codon 476 (subsequently referred to as G476 isolates). By contrast, the second group contained 14 isolates that appeared less sensitive; molecular analyses showed these isolates carried the CYP51 substitution G476S (subsequently referred to as G476S isolates). Welch's unpaired *T*‐test revealed highly significant differences in sensitivity to prothioconazole‐desthio *in vitro* between *P. biglobosus* G476 (mean EC_50_ 0.006 μg mL^−1^, SD = 0.002 μg mL^−1^) and *P. biglobosus* G476S (mean EC_50_ 0.044 μg mL^−1^, SD = 0.020 μg mL^−1^) isolates (*t*(22) = 5.70, *P* < 0.01) (Fig. 3). Thus, *P. biglobosus* G476S isolates were 7.3‐fold less sensitive than *P. biglobosus* G476 isolates. *P. biglobosus* G476S isolates were widely geographically distributed throughout Poland (Fig. 1(B)).

**Figure 3:**
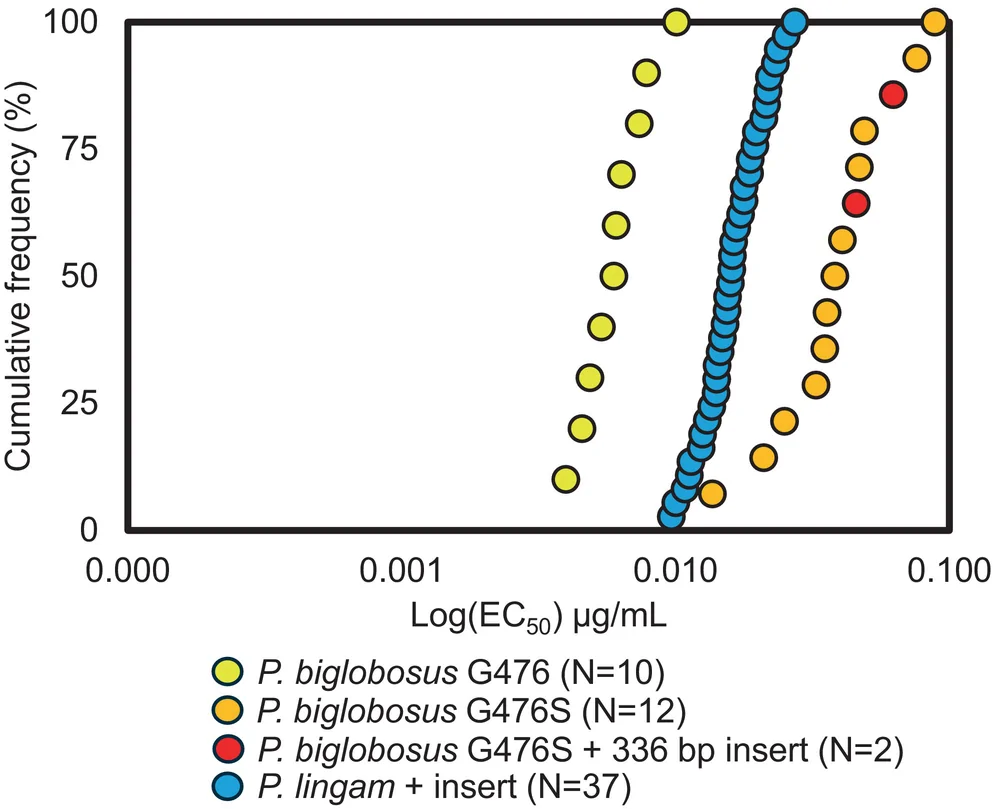
*In vitro* sensitivity of *Plenodomus lingam* (all carrying *CYP51* inserts) and *Plenodomus biglobosus* (*CYP51* gene encoding either G476 or G476S) isolates collected from Poland in 2024 to the sterol demethylation inhibitor (DMI) prothioconazole‐desthio. Note that the two G476S *P. biglobosus* isolates carrying promoter inserts (336 bp) are also indicated. Isolates are ranked according to increasing effective concentration 50 (EC_50_) values (cumulative). The number of isolates in each group is indicated (*N*). Each data point shown represents the mean of two technical replicates.

#### 3.2.2 Molecular analyses

##### 3.2.2.1 Sequencing of the *CYP51* gene

Complete *CYP51* gene coding sequences (coding for 527 amino acids) were obtained for six *P. biglobosus* isolates from 2024, while almost complete sequences (encoding 519/527 amino acids) were obtained for a further nine isolates; details of the isolates sequenced and corresponding GenBank accession numbers are given in Table 1. Alignment of the 15 sequences revealed two synonymous mutations at positions 1288 (C/T) and 1588 (A/G), neither of which correlated with differences in prothioconazole‐desthio sensitivity. A single non‐synonymous mutation was also identified at position 1442 (A/G). The position numbering for these mutations is based on the complete sequence obtained for isolate 24‐43‐7 (GenBank accession PV013628). The single non‐synonymous mutation identified corresponded to substitution G476S (numbering based on reference to the archetype sequence *Z. tritici* ^30^) resulting in a switch from glycine (G; codon GGT) to serine (S; codon AGT). Of the 15 *P. biglobosus* isolates for which the *CYP51* gene was sequenced, 8 were G476 and 7 were G476S (Table 1).

##### 3.2.2.2 PCR diagnostics for G476/G476S discrimination

Use of primers PbCommon2 and PbGly2 amplified a 99 bp product only for G476 isolates, whereas primers PbCommon2 and PbSer2 amplified a 99 bp product only for G476S isolates (Table 1; Fig. 4). The fact that all isolates were PCR screened simultaneously with both diagnostics and only ever produced an amplicon with one or the other, plus that the genotype inferred using the new diagnostics matched exactly results obtained by sequencing, confirmed the robustness of the assays. Of the 24 *P. biglobosus* isolates screened with the new diagnostic assays, 10 (42%) were G476 and 14 (58%) were G476S. Furthermore, although very faint bands were observed in testing with a *P. lingam* isolate, indicative of minor non‐specific amplification for this closely related species, these were of a larger size to the diagnostic 99 bp amplicon.

**Figure 4:**
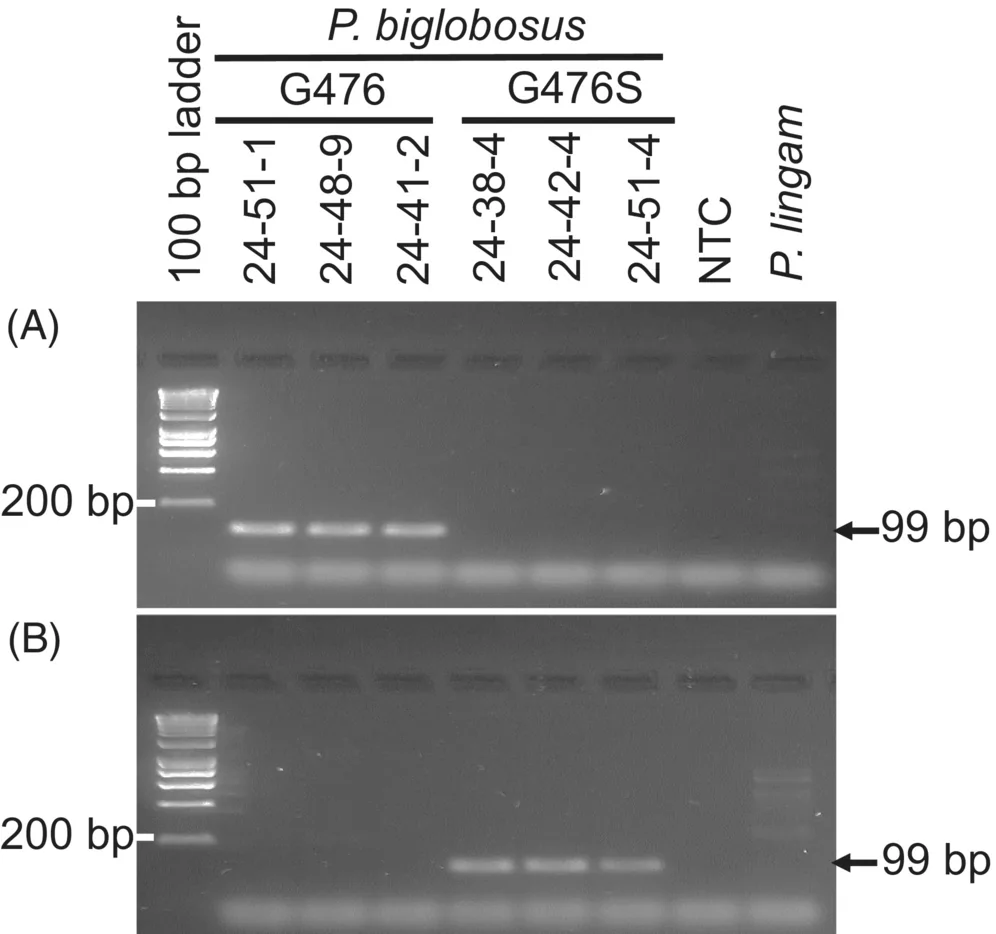
New polymerase chain reaction diagnostic assays based on *CYP51* gene sequence variation for rapid typing of G476 and G476S isolates of *Plenodomus biglobosus*. (A) Primers PbCommon2 and PbGly2 amplified a 99 bp product only for G476 isolates. (B) Primers PbCommon2 and PbSer2 amplified a 99 bp product only for G476S isolates. *Plenodomus biglobosus* isolates tested and their genotypes are given to the top of the gel, with full information given in Table 1. Lane labelled ‘NTC’ was a no‐template water control; lane labelled *P. lingam* was Polish isolate 24‐40‐4. Molecular marker sizes are indicated to the left, with an arrow showing the expected 99 bp PCR amplicon to the right.

##### 3.2.2.3 Screening of *P. biglobosus* isolates for *CYP51* promoter inserts

For 22 of 24 (92%) *P. biglobosus* isolates screened, a similarly sized amplicon of ~805 bp was obtained using primers KK2F/R (Table 1; Fig. 5). Primer KK2F was designed to target 691 bp upstream of the predicted *CYP51* gene start codon (promoter region sequence), with primer KK2R targeted 114 bp downstream of the start codon (gene coding sequence). Thus, for these 22 isolates, there was no clear evidence for *CYP51* promoter inserts. The sequence obtained for the amplicon from isolate 24‐44‐3 (GenBank accession PV013636) yielded a high‐quality sequence of 714 bp (647 bp was predicted promoter region sequence and the remainder predicted to be gene coding sequence). For the remaining 2 of 24 (8%) isolates, a larger amplicon of ~1.15 kb was amplified, which is indicative of possible inserts into the *CYP51* promoter region. Sequencing of amplicons obtained for isolates 24‐38‐4 and 24‐44‐6 (GenBank accession PV013637) revealed that both carried an insert of 336 bp (two copies of a 168 bp segment of the promoter region itself), which was located 107 bp upstream of the *CYP51* gene start codon. The geographical distribution of *P. biglobosus* isolates with or without *CYP51* promoter inserts is shown in Fig. 1(B). The two isolates carrying promoter inserts were both G476S, and visual inspection of the data (Table 1) suggested no immediately obvious differences in prothioconazole‐desthio sensitivity between them and other G476S isolates for which no promoter inserts were detected.

**Figure 5:**
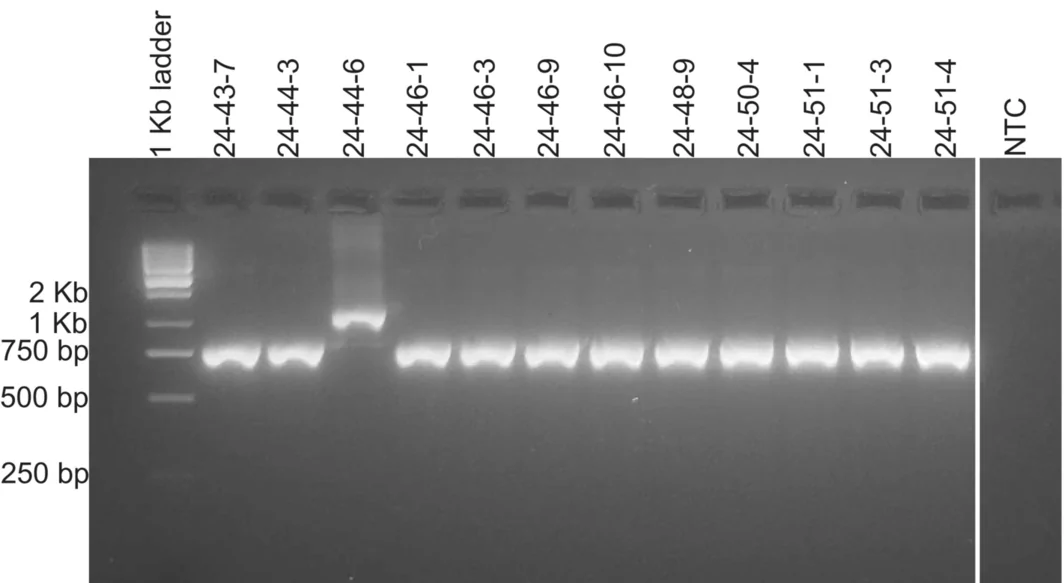
Amplification of the *CYP51* promoter region of 12 representative *Plenodomus biglobosus* isolates collected from Poland in 2024. Eleven of the isolates shown amplified an ~805 bp product indicative of no promoter inserts, while the remaining isolate shown (24‐44‐6) amplified an ~1.15 kb product consistent with the presence of an insert. Amplification of the *CYP51* promoter region used polymerase chain reaction primers KK2F/R. Isolate codes are indicated above well lanes, with full information on isolates given in Table 1. Lane labelled ‘NTC’ was a no‐template water control. Molecular marker sizes are indicated to the left.

### 3.3 Comparative fungicide sensitivity of *P. lingam* and *P. biglobosus*

The results described here are visualised in Fig. 3. Screening for prothioconazole‐desthio sensitivity showed that when ranked from the lowest to the highest, mean EC_50_ values (with SD given in parentheses) were: 0.006 (0.002) μg mL^−1^ (*P. biglobosus* G476, *N* = 10); 0.017 (0.004) μg mL^−1^ (*P. lingam* with inserts, *N* = 37); and 0.044 (0.020) μg mL^−1^ (*P. biglobosus* G476S, *N* = 14). Welch's one‐way ANOVA results suggested that significant differences in prothioconazole‐desthio sensitivity existed between one or more pairs of the three groups (*F*[2, 25.7] = 81.57, *P* < 0.01). *Post hoc* comparisons using Games–Howell tests revealed that significant differences (all at least *P* < 0.05) occurred in all pairwise comparisons between the three groups. *Plenodomus biglobosus* G476S substitution isolates were significantly less sensitive (7.3‐fold) than *P. biglobosus* G476 isolates. Compared with *P. lingam* isolates carrying inserts, *P. biglobosus* G476S isolates were significantly less sensitive (2.6‐fold), contrasting with *P. biglobosus* G476 isolates that were more sensitive (2.8‐fold).

## 4 DISCUSSION AND CONCLUSION

In this study, 37 *P. lingam* isolates collected in 2024 from across a broad geographical range in Poland were significantly less sensitive (fourfold) to prothioconazole‐desthio *in vitro* compared with five Polish isolates collected more than 20 years before (before 2002). PCR screening for *CYP51* promoter inserts revealed all Polish *P. lingam* isolates collected in 2024 carried an insert that was absent in the five older reference isolates. The sensitivity shifts identified in the Polish *P. lingam* population (collected 2024) were broadly consistent with isolates reported previously from Australia and Europe carrying promoter inserts.^19^, ^24^ Indeed, the Polish *P. lingam* population was not significantly different from German, Irish and UK populations (collected 2022/2023) in which *CYP51* promoter inserts were present at high frequencies of >85–100%, but was significantly less sensitive than the French population in which such inserts were not found.^19^ Based on visual inspection of PCR amplicons of the *CYP51* promoter region when resolved via agarose gel electrophoresis, all 37 Polish *P. lingam* isolates carried promoter inserts, in most instances producing an amplicon of a very similar size to that of a −99, ins237 reference isolate, this genotype being confirmed by sequencing of one representative isolate. This 237 bp *Sahana* TE insert also appears predominant in *P. lingam* populations from the Czech Republic, Ireland, Germany and the UK.^19^, ^20^ However, it is noted that one Polish *P. lingam* isolate yielded a significantly larger amplicon, and was instead found to be the −382, ins360 variant, previously only reported from two UK isolates.^19^ Given that the least sensitive *P. lingam* isolate from Poland (24‐42‐8) was only 6.75‐fold less sensitive to prothioconazole‐desthio, the possibility of *CYP51* target site mutations was not investigated further in this study. This research provides the first evidence for decreased DMI sensitivity *in vitro* in the Polish *P. lingam* population, associated with *CYP51* promoter inserts, although it is noted that the practical implications of these findings in terms of phoma disease management under field conditions require further investigation. Such a shift was not surprising given the extensive use of DMIs as plant protection products in Poland.^31^ Similar extensive use of DMIs is observed in some other European countries like the UK.^14^ Moreover, there is evidence that the degradation of tebuconazole, a leading DMI active compound is very slow in Polish mineral soils with low organic carbon content, and its adsorption rate is even lower in subsoils, which differentiates soils in Poland from some others in Europe.^32^ All of this could potentially have led towards selective pressure for decreased DMI sensitivity.

Initial inspection of sensitivity data obtained for the 24 modern Polish *P. biglobosus* isolates revealed that some were less sensitive than others to the DMI prothioconazole‐desthio. Therefore, we first investigated whether the observed differences could be explained by mutations in the *CYP51* gene, given that such mutations have been linked to decreased DMI sensitivity in many other fungi,^33^, ^34^ although not yet for either *P. lingam* or *P. biglobosus*.^19^, ^22^, ^23^ High‐quality *CYP51* gene sequences were obtained for 15 Polish *P. biglobosus* isolates. One non‐synonymous substitution was identified at codon 476 (numbering based on Mair *et al*.^30^) in some of the *P. biglobosus* isolates. Based on this sequence data, in combination with PCR typing data of all Polish *P. biglobosus* isolates using new G476/G476S molecular diagnostics (discussed further below), ten more‐sensitive isolates had codon GGT at position 476 that translated to the amino acid glycine and considered wild‐type (G476). Conversely, 14 less‐sensitive isolates instead had codon AGT, which instead encoded the amino acid serine (G476S). Statistically, G476 and G476S isolates were significantly different in sensitivity to prothioconazole‐desthio, with the latter being 7.3‐fold less sensitive. The equivalent substitution has been associated with decreased DMI sensitivity in many other fungi of agricultural and clinical importance, including the human pathogen *Candida albicans* in which the mutation alters the heme binding environment, with decreased DMI binding affinity and reduced enzyme activity.^34^, ^35^, ^36^ These results represent the first evidence of a *CYP51* mutation associated with decreased DMI sensitivity in *P. biglobosus*, which had not previously been identified by sequencing of older (2012/2013) UK isolates of this species.^23^ However further work is now required to investigate the role of G476S, including testing for possible incomplete cross‐resistance to different DMIs, in addition to functional and modelling studies.

Based on data obtained in this study, the G476S genotype was found to have a frequency of 58% in the Polish pathogen population (with G476 and G476S co‐occurring at some sites) and is widely distributed geographically there. Initial sequencing of the *CYP51* gene of two additional UK *P. biglobosus* isolates by the authors of this study, both of which had been collected from OSR in 2023 (described in King *et al*.^19^), revealed that one Essex isolate (23ESXLB01; prothioconazole‐desthio EC_50_ of 0.021 μg mL^−1^; GenBank accession PV013635) was G476, whereas one Norfolk isolate (23DERELB01; prothioconazole‐desthio EC_50_ of 0.028 μg mL^−1^; GenBank accession PV013634) had codon AGT translating to substitution G476S. Thus, albeit based on only a single isolate to date, the geographical distribution of G476S now also includes western Europe. Further work is now required to explore spatial and temporal dynamics of G476S, or indeed any other potential *CYP51* mutations that may be present, in *P. biglobosus* populations, particularly in the key OSR‐producing regions of Australia, Canada, China and Europe.^8^, ^37^ New PCR diagnostics have been developed in the current study for rapid detection/discrimination of G476 (primers PbCommon2/PbGly2) and G476S (primers PbCommon2/PbSer2) *P. biglobosus* isolates that can be utilised in such surveys. However, caution is required in the use and interpretation of these diagnostic assays because different mutations within the *CYP51* gene, either at codon 476 or elsewhere, might be overlooked if using these diagnostics alone.

Given that inserts (in Europe, predominantly the 237 bp *Sahana* TE) in the *CYP51* upstream promoter region have previously been associated with decreased sensitivity in *P. lingam* ^19^, ^21^, ^24^ this possibility was also investigated for *P. biglobosus*. DNA extracted from all 24 modern Polish *P. biglobosus* isolates were screened using primer pair KK2F/R,^19^ and were expected to yield an 805 bp amplicon for wild‐type *P. biglobosus* isolates (comprising a sequence 691 bp upstream and 114 bp downstream of the predicted *CYP51* start codon). Most of the *P. biglobosus* isolates tested (22 of 24; 92%), produced an identically sized amplicon of ~805 bp, as determined by agarose gel electrophoresis. This result was confirmed by bidirectional sequencing of the amplicon obtained for a single representative isolate (22‐44‐3) and demonstrated 100% identity to GenBank accession FO905635. Given that the vast majority of 2024 Polish *P. biglobosus* isolates that exhibited a broad range of sensitivities to prothioconazole‐desthio all yielded a similarly sized amplicon of ~805 bp, *CYP51* promoter inserts are unlikely to explain *P. biglobosus* fungicide sensitivity shifts identified in this study. These findings are consistent with those of King *et al*.^19^ who found no evidence for *CYP51* promoter inserts in PCR screening of 17 European *P. biglobosus* isolates collected between 2021 and 2023.

Nevertheless, it is noted that a larger product of ~1.15 kb was amplified for 2 of the 24 (8%) Polish *P. biglobosus* isolates (both G476S) in screening with primers KK2F/R, indicative of possible insertion into the *CYP51* promoter region. Bidirectional sequencing of amplicons revealed that this promoter region alteration (−107, ins336) was due to an insertion of two repeat copies (2 × 168 bp) of a segment of the promoter region itself. This contrasts with *P. lingam* in which *CYP51* promoter inserts linked to decreased sensitivity are TEs, in Europe predominantly the −99, ins237 *Sahana* TE.^19^ This finding represents the first report of *CYP51* promoter inserts in *P. biglobosus*, although it is noted that the prothioconazole‐desthio sensitivity of these two G476S isolates with inserts (EC_50_ values of 0.046 and 0.063 μg mL^−1^) was similar to that of the other 12 G476S isolates for which no *CYP51* inserts were identified (mean EC_50_ = 0.042 μg mL^−1^). In previous studies with other fungal pathogens the presence of target site alterations in combination with promoter inserts has led to far greater decreases in sensitivity than for either mechanism alone^33^, ^34^ but this does not appear to be obviously the case here for *P. biglobosus*. Further work is required to investigate whether this 336 bp insert is associated with *CYP51* overexpression and decreased DMI sensitivity, such as has been reported previously in *P. lingam*. Overall, however, *CYP51* promoter inserts do not appear to explain the decreased DMI sensitivity observed in the current study, which instead correlates well with the CYP51 substitution G476S. Ongoing monitoring of *P. biglobosus* populations for inserts is required, although it is possible, however, that such TE *CYP51* inserts are more likely to occur in *P. lingam* than *P. biglobosus* given that the former has a heavily TE‐invaded genome (32.4%) compared to the latter (<4%).^29^

Previous studies have suggested how fungicide applications might affect the *P. lingam*/*P. biglobosus* population structure given differing sensitivities of the species to fungicides^25^, although other factors such as deployment of host resistance genes (effective against *P. lingam* but not *P. biglobosus* ^13^) also need to be considered. Both of these species have the potential to be economically important OSR pathogens,^8^ so both species need to be targeted for sustainable phoma management.^13^ King *et al*.^19^ report that *P. lingam* isolates carrying the 237 bp *CYP51* promoter inserts, the predominant variant in most European populations tested, were fivefold less sensitive to prothioconazole‐desthio *in vitro* than isolates lacking such inserts. In this study, *P. biglobosus* isolates of G476S genotype were 7.3‐fold less sensitive compared with G476. Statistically, based on the Polish data set obtained in this study, *P. lingam* isolates carrying *CYP51* promoter inserts were significantly less sensitive to prothioconazole‐desthio than *P. biglobosus* G476 isolates (2.8‐fold), but significantly more sensitive than *P. biglobosus* G476S isolates (2.6‐fold). There is evidence that the equivalent substitution in the clinical pathogen *C. albicans* is not costly in fitness terms, at least under laboratory conditions.^38^ If the G476S substitution in *P. biglobosus* carries no fitness penalty, it is possible that this genotype will increase over time under selection by DMI fungicides. Further work is required to investigate whether *P. biglobosus* (carrying G476S) may be selected for over *P. lingam* (carrying *CYP51* promoter inserts) under field conditions via application of DMI fungicides. Such studies should also consider other factors likely to influence pathogen population structure, particularly the deployment of host resistance targeted only to *P. lingam* and its current effectiveness.^13^

Ongoing proactive monitoring of both *P. lingam* and *P. biglobosus* populations for any future DMI sensitivity shifts, and identification of the underpinning molecular mechanisms will be important as a component of fungicide resistance management strategies. Further work for both species is required to investigate the possibility of enhanced fungicide efflux via overexpression of efflux pumps, which was not tested in this study. It is possible that *P. lingam* may in future develop *CYP51* target site mutations, perhaps more likely those encoding G476S, given that this substitution has now been confirmed in *P. biglobosus*. Alternatively, *CYP51* promoter inserts leading to target site overexpression and decreased DMI sensitivity may emerge in *P. biglobosus*. Should increasingly complex *CYP51* variants emerge for either species that encompass both these resistance mechanisms, they are likely to exhibit far greater decreases in DMI sensitivity than for either mechanism alone, such as has been reported for other economically important phytopathogenic fungi.^33^, ^34^

## Data Availability

The data that support the findings of this study are available from the corresponding author upon reasonable request.
